# Biologising parenting: neuroscience discourse, English social and public health policy and understandings of the child

**DOI:** 10.1111/1467-9566.12223

**Published:** 2015-02-12

**Authors:** Pam Lowe, Ellie Lee, Jan Macvarish

**Affiliations:** 1 School of Languages and Social Sciences, Aston UniversityBirmingham, UK; 2 SSPSSR Cornwallis Building, University of KentUK; 3 CHSS, University of Kent

**Keywords:** neuroscience, parenting, policy analysis, child development

## Abstract

In recent years, claims about children's developing brains have become central to the formation of child health and welfare policies in England. While these policies assert that they are based on neuro-scientific discoveries, their relationship to neuroscience itself has been debated. However, what is clear is that they portray a particular understanding of children and childhood, one that is marked by a lack of acknowledgment of child personhood. Using an analysis of key government-commissioned reports and additional advocacy documents, this article illustrates the ways that the mind of the child is reduced to the brain, and this brain comes to represent the child. It is argued that a highly reductionist and limiting construction of the child is produced, alongside the idea that parenting is the main factor in child development. It is concluded that this focus on children's brains, with its accompanying deterministic perspective on parenting, overlooks children's embodied lives and this has implications for the design of children's health and welfare services.

## Introduction

In recent years, claims about children's developing brains have become central to the formation of child health and welfare policies in England. Moreover, this has included a strong shift towards the construction of parenting as a key determinant of brain development and thus the child's future. While these policies assert that they are based on neuro-scientific discoveries, their relationship to neuroscience has been debated (see for example Wilson 2002). This trend is part of the broader shift to ‘neuroculture’ in which interpretations of the brain are reconstructing existing understandings of mind/body, nature/culture, and identity/development (Vidal 2009, Williams *et al*. 2012). Within child health and welfare policies, we will argue that ‘neuroculture’ has led to a particular portrayal of children and childhood, one that is marked by a lack of acknowledgment of child personhood. In addition, within the resulting parental determinism, the emphasis on intervention produces a specific idea of children ‘at risk’ thus justifying a culture of surveillance and monitoring in relation to family life.

This article arises from a wider study of the emergence of ‘neuroculture’ in parenting policies.^1^ The study was not a critique of neuroscience itself, but sought to investigate through documentary analysis the ways in which ideas about children's brain development were shaping the policy context. Within this we considered the construction of the child within policy discourse, in particular, the significance attributed to parental actions in developing the child's brain for good or bad. This article will describe how the child^2^ is represented within these claims and explore their implications for children and their parents more generally. We will argue that despite policy claims that the child is at the centre of policy thinking and that ‘every child matters’, the research has shown that by focusing on the brain, a highly reductionist and limiting construction of the child is produced which has implications for the design of health and welfare services.

## The rise of ‘neurocultural’ claims

As many authors have documented, while neuroscience and brain-imaging techniques have future potential to assist in the development of scientific insights, many of the current claims about neuroscience in popular arenas are not based on evidence (see for example, Racine *et al*. 2010, O'Connor *et al*. 2012, Satel and Lilienfeld 2013). As Rose and Abi-Rached 2013 argue (2013) while policymakers may look to neuroscience for answers to issues such as enhancing child development or reducing anti-social behaviour, to date it has little answers. Moreover, the areas of policy now using brain development to give weight to their claims are often those with a longer history of similar interventions. Rose and Abi-Rached (2013: 162) suggest that while the neuroscientific arguments may be being used to give policy initiatives ‘a sheen of objectivity’, they are also an important indicator of a shift of broader understandings about the brain.

An important element is that often in wider discourses the limits of neuroscience are frequently overlooked. As Roskies (2006) argues, neuroscience can show the mechanisms for behaviour, but it does not show that the brain determines behaviour. Fine et al (2013) maintain that over simplified and rigid brain-to-behaviour narratives fail to account for the interplay of social and environmental factors. Yet as Satel and Lilienfeld (2013: 15) suggest, within some neuro-discourses, the mind and the brain are coming to be seen as identical rather than interrelated:

Brain-based explanations … for social behaviours that elude crucial psychological, social, and cultural levels of analysis fall into the trap of neurocentrism … The brain enables the mind and thus the person. But neuroscience cannot yet, if ever, explain how this happens.

In other words, while neuroscience has the capacity to bring new understandings about brain functioning and aid the development of understandings of brain conditions such as dementia, it cannot be used to explain humanity more broadly (Tallis 2012).

## ‘Neurocultural’ claims and children's brains

In relation to children, much of the focus within brain-based understandings constructs the early years as a time of unique neural vulnerability, when without the correct environmental influences, normal development will be disrupted and may never be recovered (Thornton 2011). Bruer (1999) has argued that these claims have arisen from three particular ideas associated with brain development in the first three years that have been misconstrued. The first idea is that the developmental synaptogenesis (rapid increase in synapse density) that takes place is uniquely significant. Bruer (1999) argues that the early years are not the only time for developmental synaptogenesis and more synapses do not necessarily mean more brain functioning. The second idea is the early years are a time-sensitive period for development. Although it is the case that a few areas are time-sensitive (for example, deprivation of vision during early years will affect development), Bruer (1999) points out that time-sensitive skills or behaviours are exceptions and it is an exaggeration to see the early years as a general time-sensitive period. The final area is the notion of enriched environments. Bruer (1999) argues that while very extreme neglect can negatively impact on a child's brain development, this is not the same as saying that brain development can be enhanced by enriching environments in the early years.^3^

The notion that the brains of neglected children are at risk is worth considering further. While it is the case that very extreme circumstances can lead to altered brain development (Behan *et al*. 2008), the issue is complex. The issue of deprivation and neglect has been extensively explored following the discovery of children raised in Romanian Orphanages in the 1990s where large numbers of malnourished children were found confined to cots within institutions with minimum adult contact and little to no sensory stimulation (Rutter *et al*. 1998). Many of these children were subsequently adopted into Western countries, and their progress has been tracked. Rutter and colleagues' (2010) research found that there could be good recovery even after this extreme early deprivation and that it was institutional deprivation, rather neglect itself, which was a key determinant of development issues. In addition, while the work of Perry (1997) is often used to justify brain-based approaches, he has always been clear that there is no automatic route between emotionally neglected children and adult violence.

## Policy and the developing child

Yet despite the neuroscience understanding that the brain is actually notable for its ‘plasticity’ and it is adaptable rather than ‘hard-wired’ (see Pascual-Leone *et al*. 2005 for a discussion) this article will show that this has not stopped policymakers from adopting and incorporating deterministic ‘brain claims’ into English social and public health policy. In England, the claims began to be noticeable in the early 2000s, but since then there has been an expansion of the utilisations of them. The move to promote and adopt early intervention child health and welfare programmes (e.g. Allen and Duncan Smith 2008) is a notable example of this trend. These early intervention policies differ from previous social initiatives in that they are premised on the specific ideas arising from ‘neuroculture’ outlined above. They take as their starting point the notion that the ‘early years’ are the most important in a person's life because of an irreversible role in cognitive and emotional development and thus early intervention is justified and necessary (Macvarish *et al*. 2014).

In the earliest years of life, the ‘environment’ is usually understood to be the family or the mother, hence the intense focus on parenting and motherhood. Indeed as O'Connor and Joffe's (2013) study showed, the intra-uterine environment is increasingly named in media stories on brain and child-development. Media stories claim that what happens in the womb influences the future potential of a child in a range of areas from the risk of psychiatric disorders to sexual orientation (O'Connor and Joffe 2013). Hence early intervention policies are situated within a broader ‘neurocultural’ discourse that focuses on parenting as both a cause of and solution to the child's brain development. This deterministic view of child development fits into wider understandings of children's lives.

Turmel (2008) argues that the developmental model of childhood emerged at the turn of the twentieth century. He argues that it was the nineteenth century move for the categorisation and classification of children though observation and measurement which led to a development of the concept of the ‘normal child’ (Turmel 2008: 77). This concept is reflected in an understanding of child development as a series of universal stages which can be seen in the work of Piaget (Hill and Tisdall 1997, Turmel 2008). Turmel (2008) shows that while at first monitoring children was seen as a role for mothers, the site of observation shifted to experts with the founding of child-welfare clinics such as that opened by Pritchard in London in 1907. It is into this long history of child development, parenting advice and child-welfare initiatives that the current policy initiatives need to be situated.

As Corsaro (2011) has outlined, there has been widespread criticism of the universal deterministic model of child development which fails to consider the ways in which children shape their own lives within the social and economic structures that they live in. Moreover even young children can be competent and articulate about their thoughts and feelings, providing they are asked in the right way (Alderson 2013). More recent developmental theories have often focused on the internalisation of adult skills and knowledge (James *et al*. 1998; Corsar, 2011), yet this understanding of children as social actors, albeit within a world usually shaped by adults, is rarely acknowledged. Consequently, it is not much of a leap between the notion that children are predestined to ‘learn’ the skills from adults to the idea that the brain itself needs to be ‘wired’ appropriately, and thus children's futures lives are predestined by early years brain development. We will argue that it is this notion of the brain rather than the mind that disembodies the child within health, welfare and parenting policies.

Wastell and White (2012) argue that this presumption of biological determinism and its associated medicalisation has had profound effects on child welfare, and led to a shift in emphasis to standardised targeted interventions to ‘cure’ the problem. Featherstone *et al*. (2013) have also raised concerns about the implications of this shift in policy and the links to changed understandings of child protection rather than family support. Both these papers draw attention to how the child is both biologised yet disembodied. The brain is thus becoming central to the identification of children ‘at risk’ and interventions targeted to manage this perceived biological lack. Moreover, as Hulbert (2004) has documented in the US, the brain is increasingly seen ‘as the child’ rather than being part of the child. Thus a normalised view of brain development based on a rigid understanding of child development leaves little room for children to be considered as autonomous individuals with the capacity to shape their own futures.

Moreover, understanding the architecture of the brain, tells us little about thoughts and feelings as mental activities do not, and probably never will, map easily onto brain regions (Satel and Lilienfeld 2013). If the brain is the sole or major determinant of responsibility for thoughts and actions, then any presumption of free-will could be seen to be illusionary (see Murphy and Brown, 2009 for a fuller discussion). Yet despite widespread critiques that a reductionist position is not warranted, the idea that brain development determines the mind, and thus future behaviour is increasingly central to child health and welfare policies. Indeed a key rationale for early intervention is that some parents are incapable of developing their children's brains ‘properly’ (Rose and Abi-Rached 2013). In other words, ideas of intergenerational transmission of ‘good’ and ‘bad’ behaviour through parenting is central to brain-based policy.

## Methodology

This article is developed from a wider study concerned with the emergence and embedding of ‘brain-based’ claims in British policy concerned with ‘parenting’. The object of the research was to understand the ways in which ‘parenting’ has been constructed as a policy problem, in particular, one which can be resolved through the adoption of ‘scientific’ approaches to its improvement. It was seeking to explore the adoption of concepts and vocabulary attributed to neuroscience as a particular focus in order to study what appeared to be an explicit ‘scientisation’ of the parent-child relationship.

The project sought to trace the adoption of claims using the authority of neuro-scientific evidence in policy-thinking from 1998 to 2012, through analysis of a purposive sample of 42 documents. These documents were selected by the research team in line with the criteria detailed below. We identified documents which were central to the formation of parenting policy across a number of domains. As well as those directly on parenting and early child development, the full sample included those looking at social exclusion, criminal justice and health and maternity services. Following an initial screening, 42 documents were chosen for full analysis based on the inclusion of key concepts (e.g. parenting, brains, attunement) and/or their influence on later policy. We thus sought to uncover a chronological account that mapped how brain discourses moved from being a ‘backstage’ discussion in early policies to a significant way of organising the relationship between health and early years practitioners, the parenting workforce and families.

Included in the sample were documents published by government departments including consultation and strategy documents (29), reports commissioned by government departments (7), as well as those emanating from advocacy groups (6) which have subsequently become key reference points in later policy. Full details of the sample can be found in Lee *et al* (2013). All the documents were uploaded into NVivo (QSR International, Melbourne) and then thematically analysed, through close reading and coding of documents. These codes were then used to identify key themes. We also sought to identify the links between the different documents by tracing how understandings of the different concepts travelled through different documents over time. In addition we undertook some mapping of key terms (e.g. parenting, brain, cortisol) using the word count functions to get a sense of how the policy discourse was changing over time.

This article uses examples from our sample as representative of the broader trends we have found in the study. Quotations have been identified which illustrate how the two key areas: the eclipsing of the mind into the brain, and the brain rather than an embodied child, are deployed in parenting policies. This will illustrate the ways in which brain-determinism in parenting policies leads to a narrow conceptualisation of children and child health within which the child is simultaneously both central to the policy agenda, yet overlooked in terms of their personhood.

## Mind-less brains

The idea of intergenerational disadvantage has a long history in English welfare policies, and a common theme among family welfare policies is the notion of ‘breaking the cycle’. Cunningham (2005) has documented this history of interventions to ‘save’ children from incompetent or indifferent parents. What is new, however, is the idea that brain development is the key to achieving this (Rose and Abi-Rached 2013). For example, *Every Child Matters*^4^ (Chief Secretary to the Treasury 2003) emphasised the role of parenting as a key site of potential intervention in children's lives, but made no mention of brain development. A year later, brain development is mentioned in *Support from the Start*^5^ (Sutton *et al*. 2004). This report highlights a number of areas of risk factors for the development of behavioural issues in children and does mention the possibility of adverse brain development caused by stress (referencing the work of Perry). However, it argues that a combination of physiology and social factors could lead to aggressiveness (Sutton *et al*. 2004). Less than a decade later, the idea that brain development is a key determinate seems to be uncritically accepted in a wide range of reports. For example in *Healthy Lives*, *Healthy People*^6^ (Department of Health 2010: 18) the role of neurons is central in this extract:

At birth, babies have around a quarter of the brain neurons of an adult. By the age of 3, the young child has around twice the number of neurons of an adult – making the early years critical for the development of the brain, language, social, emotional and motor skills.

An important element is a new idea of the process of intergenerational transmission. At its heart, is the notion that dysfunctional families fail to develop their children's brains (probably because of their own brain-dysfunction). By intervening early enough in young children's lives, the policy can ensure that the future children within these families have better opportunities for brain development and thus will be able to optimise their own children's lives in future generations. Allen and Duncan Smith's (2008)^7^ report is typical of this approach. It focuses on children's brain development in the early years to justify a strategy of intervention:

The Early Intervention objective is nothing less than to replace a vicious cycle with a virtuous circle; to help every child become a capable and responsible parent who in turn will raise better children who themselves will learn, attain and raise functional families of their own. (Allen and Duncan Smith 2008: 5).

This report claims that early intervention can reduce the ‘flow’ of people into the dysfunctional ‘stock’ (Allen and Duncan Smith 2008: 24). Although it does not overtly argue that we need to limit the number of children born (except teenage pregnancy), it does seek to stop the transmission of ‘undesirable behaviours’. Within this report there are a remarkable number of social issues that claimed to be potentially preventable should children's brains be built properly. These include unemployment, criminality and drug-addiction (Allen and Duncan Smith 2008: 12). Indeed the entire economic rationale for the policy is that it will reduce future expenditure on welfare, health and criminal-justice services (Allen and Duncan Smith 2008: 15). Thus child brain development is a determinist feature of future behaviour, and parenting interventions are necessary as the (incompetent) parent's brains may not have developed sufficiently for them to be able to raise their children adequately without intervention.

What is interesting is the ways in which the mind, the thinking and feeling consciousness of individuals, is both absent and present within policy documents. The emphasis on the brain as central to the (good) functioning of the developing child, is portrayed as determined and a matter of appropriate neuron-development. So for example in *Reaching Out: An Action Plan on Social Exclusion*^8^ it is stated that:

It is also known from research just how important a child's early experiences are to the development of the brain. The child who is spoken to will develop speech and language neural systems, and the child who has motor practice and exploration opportunities will develop neural systems which allow walking, running and fine motor control. The child who is nurtured and loved will develop the neural networks which mediate empathy, compassion and the capacity to form healthy relationships (Cabinet Office 2006: 47).

In this extract we can see how the capacity of the mind to be thoughtful and have feelings is reduced to particular neuron pathways, but it goes further than that. It uses an image of very extreme neglect to justify the position. The idea that some children are never/rarely spoken to and are tethered rather than being able to move is reminiscent of the conditions of the Romanian orphanages which goes beyond issues of deprived households and social exclusion.

The notion that empathy is a skill rooted in neurological development in early childhood can also be found in other policy documents. For example in *The Child Health Promotion Programme: Pregnancy and the First Five Years of Life* (Department of Health 2008)^9^ the section entitled ‘Promotion of social and emotional development’ starts with the statement that:

More is known today than ever about the neurological development of infants, and the impact of poor attachment and negative parenting on a child's physical, cognitive and socio-emotional development – not only in childhood, but also as a key determinant of adult health (Department of Health 2008: 23)

The section does not mention any other influence suggesting that neurological development is the sole or main cause. Later on the same page it highlights that successful parenting support includes:

The ability to promote attachment, laying the foundations for a child's trust in the world, and its later capacity for empathy and responsiveness (Department of Health 2008: 23)

Taken together the statements in this report again suggest that the emotional intelligence of adults arises from brain development in early childhood. Clearly there are differences between children and groups of children in regards to emotional development. However the suggestion that these are reducible to brain development in early childhood ignores factors such as social and educational experiences (and resources) way beyond parent-child interactions. Indeed the meaning of ‘empathy’, ‘compassion’ and ‘healthy relationships’ are cultural and historical questions and the reduction of these to neural networks foregrounds brain-architecture at the expense of an understanding of the mind as thoughtful.

This policy premise that child brain development fixes the capacity to be empathetic and compassionate also seems to be undermined by the notion that parents can be trained to be ideal parents. If the brain development in childhood is deterministic, then it would seem reasonable to suggest that no amount of training could re-wire the adult brain. Yet a significant proportion of parenting policy is designed to ensure that inadequate parents learn to deliver appropriate emotional responses. For example Allen (2011: 17)^10^ sets out the case for parenting interventions to develop the attunement of parents and young children. Attunement is deemed to be the ability to be able to respond to a child's emotional needs and without it children will lack empathy and have a propensity towards violence. The report emphasises that parenting interventions can assist to develop these skills, including an emphasis on non-violent parenting. Thus the propensity for violence seems to be both ‘hard-wired’ into children yet malleable in adults, a contradiction that is not easily explainable.

Yet what is clear in these brain-based policies is that the mind of the developing child is largely absent from the debates. Little consideration is given to the notion that children are thinking and feeling individuals who play a role in their own destinies. The ways that the brain comes to stand for the child will be developed further in the next section.

## Brain as child

As Rose and Abi-Rached (2013) have outlined, while the idea that dysfunctional families ‘hand on’ their dysfunction through generations is not new, ideas from neuroscience have been used to argue that the brain is the mechanism of the transition. Typical of the explanatory frameworks is this version stated in *The Child Health Promotion Programme: Pregnancy and the First Five Years of Life*:

A child's brain develops rapidly in the first two years of life, and is influenced by the emotional and physical environment as well as by genetic factors. Early interactions directly affect the way the brain is wired, and early relationships set the ‘thermostat’ for later control of the stress response. (Department of Health 2008: 9)

Again here we can see illustrated the emphasis on the first few years as the critical period in a child's life when the brain both develops and gets ‘set’. The other indicator here that is noteworthy is the idea that a crucial component is the ‘stress’ response. This can be linked to the psychological notion that an insecure attachment between children and their primary caregivers will go on to have implications for other types of relationships. The idea of attachment as an essential part of child development arose from the work of Bowlby and others such as Ainsworth and despite the widespread evidence critiquing this understanding; it remains prominent in ideas about child development (Harris 2007, Wall 2010). A connection is made between the stress responses in childhood and later adult stress responses. The implication is that these children will be hyper-vigilant and thus have a tendency for antisocial behaviour leading to deviant adulthood. For example, in *The Foundation Years: Preventing Poor Children becoming Poor Adults*^11^ it states:

The development of a baby's brain is affected by the attachment to their parents and analysis of neglected children's brains has shown that their brain growth is significantly reduced. Where babies are often left to cry, their cortisol levels are increased and this can lead to a permanent increase in stress hormones later in life, which can impact on mental health. (Field 2010: 41)

The idea that brain-wiring is a crucial determinate of anti-social behaviour in later life is one of the ways in which the child's brain comes to stand in as the child. Thus, it is not the child as a person that needs to be cared for in their own right, but the brain that needs proper development. For example, *Early Intervention: Good Parents*, *Great Kids*, *Better Citizens* justifies early intervention because:

Human infants arrive ready to be programmed by adults … Neuroscience can now explain why early conditions are so crucial: effectively, our brains are largely formed by what we experience in early life. (Allen and Duncan Smith 2008: 56–7)

It is only through the assumption that it is the brain rather than the child that is the key point of intervention that the focus on the ‘right’ form of parenting thus becomes a crucial determinate of child welfare. It is not necessary to reduce poverty or provide decent housing for example, as the only element that matters is the interaction between the parent and the child. Hence poverty for children is no longer measured in material terms, it is reduced to an attribute of parenting. After all, if the brain is the child, the needs and health of the body are no longer a necessary concern of the state, but just an outcome of (poor) parenting.

## Parental determinism

Consequently, parents are repositioned as both a potential cause and solution to the ‘problem’ of the developing child, and the quality of parenting, rather than the child itself, come to be a major focus of state policy. Thus, while the child is nominally at the centre of early intervention policies, the focus remains on the child as a ‘becoming’ rather than a ‘being’ (Qvortrup, 1994). In other words, early intervention policies are based on the need to develop children as future citizens rather than an understanding of children as part of the society today.

In this context, parenting can be understood as either ‘optimising’ or ‘limiting’ brain development in children. In terms of ‘optimisation’, ‘neurocultural’ ideas are deployed to underpin new insights into how we might enhance our child's brain capacity by loving and stimulating them in particular ways. In policy terms, the claims for this are about ensuring ‘normal’ development, but it is useful to remember that commercial claims of ‘brain-training’ toys and activities aimed at ‘enhancing’ child development are a constant background within wider culture (Nadesan 2002). The ‘limiting’ perspective has more pessimistic connotations, expressing anxieties about social disorder and alienated individuals but also constructing particular social groups (usually the poor) as neurologically disadvantaged and behaviourally problematic. In this invocation of brain science, the effects of inappropriate parenting are inscribed in the infant brain, bearing consequences not just for the child and its parents, but for society as a whole.

Hence within the policies, there is an increased emphasis on parent-training in order to potentially avoid these issues. Consequently parents are deemed to be a key determinate in the development of the child's brain, and thus the child itself. These ideas can be seen in the following extracts:

The brain stem of a young child growing up in an atmosphere of unpredictable violence, such as families where routine domestic abuse takes place, is likely to become over reactive, by comparison with that of a child developing in a calm environment. If that unpredictable violence persists, the young child may learn to become hyper-vigilant to perceptions of threat; and this hyper-vigilance may undermine his capacity to concentrate on ordinary childhood activities, as well as making him over-prepared to respond impulsively and aggressively. (Sutton *et al*. 2004: 34)

Although poor parenting practices can cause damage to children of all ages, the worst and deepest damage is done to children when their brains are being formed during their earliest months and years. The most serious damage takes place before birth and during the first 18 months of life when formation of the part of the brain governing emotional development has been identified to be taking place. (Allen 2011: 15)

These quotations illustrate the ways in which parenting is seen as a crucial determinate of brain development and thus determines the future potential of the child. However as Kagan (1998) has argued, the appeal of brain claims resides in the prior cultural tendency towards ‘infant determinism’ in which the early years are said to determine adult lives. Yaqub (2002) observes that the scientific vocabulary endows pre-existing commonsense ideas of infant determinism with renewed authority. As Wall (2010) and O'Connor *et al*. (2012) have shown, societal concern about the impact of early experiences on later development, developed with the popularisation of psychoanalysis and attachment theory in the early twentieth century. Ideas about brain development have now become an ‘important reference point in child-rearing decisions’ used to ‘indicate the ‘‘correctness’’ of parenting practices’ (O'Connor *et al*. 2012: 221).

Although the use of a neuro-vocabulary of synapses, neurons and cortisol might suggest that new scientific evidence demands a shake-up of existing ways of raising children, in fact, the recommendations using the authority of brain science are remarkably similar to existing ideas about what (and who) constitutes good parenting. This suggests that it is cultural norms, rather than scientific discovery, that are shaping the claims of good parenting within policy (Thompson and Nelson 2001, Wilson 2002).

## Writing children off, holding parents to account

This article has set out three trends in policies related to the design and development of services for children and their families. Emerging from the adoption of neuroscientific ideas, the (potential) brain rather than the child has become the major site of intervention. As the brain has become more prominent, embodied children have increasingly disappeared from consideration. The ability of children to think, act or feel has been reduced to an outcome of their parent's behaviour towards them within the early years. The reduction of an understanding of children to a mind-less brain, stands in opposition to any recognition of children as social actors.

In her review of policy assumptions, Mayall (2006) argued that while there had been some changes in policymaking that recognised that children have a right to be heard, there were still underlying assumptions that the value of children was largely as future adults. She also argued that child protection is often prioritised over provision for children and children's participation is often tokenistic. This analysis has shown that in these policies this trend has continued. Understandings from the sociology of childhood, such as the social construction of childhoods and that children actively contribute to their own social development within co-constructed families (Corsaro 2011), do not appear in the documents. Moreover children's position as ‘at risk’ from poor parenting continues the theme of children as victims that Mayall (2006) previously identified.

In addition, the emphasis on parenting as the key determinate of child development has other important implications for children's health and welfare. It justifies increasingly harsh welfare regimes, as poverty need no longer be considered seriously, as the main concern is with current and potential transmission of social disorder. Good parenting is said to be able to provide ‘resilience’ to adverse circumstances, and thus investment in the provision of parenting classes can be prioritised over other issues such as an adequate income or housing. As Hulbert draws out, despite the intentions of many of the early US brain advocates that this way of understanding child development would increase public funding of programmes to help children, the consequences of the way brain science has been used in the US has been a profound fatalism and pessimism:

If young brains subjected to deprived conditions, and to the inadequate parenting that often goes along with them, are irrevocably damaged – pickled in stress hormones, stripped of synapses – there is no time to waste, that is true. Yet such alarm, though it conveys urgency, can all too easily fuel defeatism. If children become neurologically unresilient at an early age, then only intrusive and intensive remedial efforts seem equal to the job. And if – or, let's face it, when – such intervention fails to materialise, the case for subsequent help is bound to seem weaker. (Hulbert 2004: 316)

The fact that this fatalism is not absolute – the child is negatively affected only if the parent fails to correctly nurture and stimulate the child – is significant. The idea that the years 0 to 3, or even minus 9 months to 3, represent a ‘critical period’ for development reduces any sense of a child's agency to a remarkably short time-frame, and it simultaneously demands of the parent the exercise of a huge amount of agency in doing the right thing for their child.

According to Nadesan (2002: 24), brain science, as popularised in the US, functions as a ‘tool of social engineering for the poor’ while also promising to ‘engineer middle-class parents’ anxiety by holding them accountable for each and every state of their infant's ‘development’’ (Nadesan 2002: 24). The project has found a similar trend in UK policy. Not only do brain claims shut down any discussion about different ways of raising children within policy understandings, they also promise to make parental love directly measurable in the behaviour of their offspring. The parent is therefore simultaneously disempowered by being denied the ability to make their own judgements about how best to raise their child while also having their love described as the architect of their child's brain, evident in the child's happiness and achievements and theoretically ‘readable’ through the technology of the brain scan. In this way, parents are held to account for an impossibly burdensome range of decisions by an apparently objective locus of authority – the brain.

Policies that seek to support families in crisis or ensure that children are protected are clearly important. Indeed if children and their parents enjoy or benefit from programmes developed from the new ‘neurocultural’ environment, this is clearly constructive. However, the deterministic accounts of brain-to-behaviour has other political consequences. It further erodes the line between children at risk and children who might become a risk to others (James and James 2008). In doing so, it justifies surveillance of (poor) families. Moreover, at the most extreme end of policy, Featherstone *et al*. (2013) have warned that in the name of protecting infant brains, state agencies are increasingly ‘intervening early’ in families deemed problematic to remove children from their birth parents into the custody of the state. In addition, it has been argued that such removals should be more rapidly converted into permanent adoptions by more suitable families to prevent permanent damage being inflicted on infant brains.

## Conclusion

This article has sought to illustrate the ways in which the development of ‘neurocultural’ discourse has led to particular construction of the child within English health and social policy. It has documented the ways in which current policy discourse is increasingly focused on brains and parents rather than being concerned with children's embodied lives. The persistence of deterministic ideas indicates that the ideology of infant and parental determinism is prior to, and stronger than, any actual evidence emanating from the scientific domain. Within this policy framework, children as embodied social actors, have increasingly disappeared and have been replaced by the child as a mind-less brain, a potential victim of poor parenting. Thus while the policy rhetoric claims to be centred on the child, the focus on the brain leads to a limited understanding of children. As Hubert (2004: 316) states it reduces the infant child to a ‘fate shaped by their parenthood’.

Clearly policy documents are different from both policy implementation and any actual impact on children and parent's lives. Nevertheless, they are an important indicator of trends in political understanding and can have implications for the resourcing of health and welfare services. Parents do provide a crucial component to children's lives, but the idea that children are just the outcome of parental actions is overly deterministic. It also ignores the important insights from the sociology of childhood that have clearly shown that children are active in the construction of their own and families lives. Moreover, rather than children being at the heart of child and family policy, brain-based claims refocuses the emphasis to parenting. The parenting/brain nexus configured in this policy discourse can be situated alongside health and welfare cuts without contradiction as it suggests that disadvantaged children need better trained parents rather than services.

## Notes

1 The project Biologising Parenting: Neuroscience discourse and English social and public health policy was funded by the Faraday Institute within the ‘Uses and Abuses of Biology’ Grants Programme.

2 While children are not a homogenous group, within policies normative assumptions are often made. We use the term ‘the child’ to reflect the singular position often depicted.

3 For a detailed discussion of the rise of the Early Years Movement and the critiques on this position please see Macvarish *et al*. (2014).

4 Every Child Matters was published as a Green Paper which is a government consultation document.

5 Support from the Start was a research report for the Department of Education and Skills.

6 Healthy Lives, Healthy People: Our Strategy for Public Health in England was a White Paper, setting out government policy.

7 Early Intervention: Good Parents, Great Kids, Better Citizens was a report from the Centre for Social Justice (an independent think tank) but authored by two Members of Parliament.

8 Reaching Out was a government report setting out strategy for the Cabinet Office.

9 This document is best practice guidance from the Department of Health.

10 Early Intervention: The Next Steps was an independent report for government authored by an MP.

11 This was an independent report for the Prime Minister authored by an MP.
